# Advances in Thyroid Function Tests: Precision Diagnostics and Clinical Implications

**DOI:** 10.7759/cureus.48961

**Published:** 2023-11-17

**Authors:** Hamd E Yazdaan, Fnu Jaya, Fnu Sanjna, Maha Junaid, Sohaib Rasool, Ahmadullah Baig, Mohammad Zubair Natt, Nikhil Maurya, Subhan Iqbal, Bisto Alungal Yeldo, Alina S Khan, Giustino Varrassi, Satesh Kumar, Mahima Khatri, Saira K Awan

**Affiliations:** 1 Internal Medicine, Khyber Medical College, Peshawar, PAK; 2 Medicine, Ziauddin University, Karachi, PAK; 3 Medicine, Shaheed Mohtarma Benazir Bhutto Medical College, Karachi, PAK; 4 Medicine, Shifa College of Medicine, Islamabad, PAK; 5 Medicine, Bakhtawar Amin Medical and Dental College, Multan, PAK; 6 Internal Medicine, Allama Iqbal Medical College/ Jinnah Hospital, Lahore, PAK; 7 Medicine, Rahbar Medical and Dental College, Lahore, PAK; 8 Medicine, Ruxmaniben Deepchand Gardi Medical College (RDGMC), Ujjain, IND; 9 Diagnostic Radiology, Dr. Ziauddin Hospital North Nazimabad, Karachi, PAK; 10 Medicine, Mari State University, Yoshkar-Ola, RUS; 11 Medicine and Surgery, Liaquat National Hospital and Medical College, Karachi, PAK; 12 Pain Medicine, Paolo Procacci Foundation, Rome, ITA; 13 Medicine and Surgery, Shaheed Mohtarma Benazir Bhutto Medical College, Karachi, PAK; 14 Medicine and Surgery, Dow University of Health Sciences, Karachi, PAK; 15 Medicine, Kabir Medical College, Peshawar, PAK

**Keywords:** clinical, hormone, trt, tsh, tft, thyroid, advances

## Abstract

This narrative review explores the evolving field of thyroid function testing, explicitly highlighting the significance of precision diagnostics and their substantial impact on clinical practice. Commencing with a comprehensive examination of the historical progression of thyroid diagnostics, the discourse proceeds to explore recent developments, highlighting the paramount importance of accuracy in testing methods. The primary issue under consideration is the crucial requirement for accuracy in the field of therapeutic practice. The review critically examines the problems related to the interpretation, standardization, and ethical considerations in examining advanced laboratory techniques, novel biomarkers, and state-of-the-art technologies like immunoassays, molecular testing, and automation. The focus on the paradigm shift towards precision diagnostics brings attention to the complex connection between test results and their direct influence on patient care. This investigation expands upon the incorporation of imaging and molecular diagnostics, highlighting the rising significance of precision in customizing treatment strategies. In summary, the study provides a prospective viewpoint, recognizing the persistent obstacles and highlighting the want for dependable, uniform methodologies in thyroid diagnostics. This narrative's primary objective is to guide physicians, researchers, and stakeholders in effectively navigating the intricate nature of contemporary thyroid function tests, with a particular emphasis on resolving the fundamental issue of precision.

## Introduction and background

Thyroid problems encompass a wide range of medical issues that impact the thyroid gland, an anatomical structure resembling a butterfly in the neck region [1]. The thyroid gland plays a pivotal role in regulating several metabolic processes in the human body, encompassing the production and consumption of energy. Thyroid-related disorders can present in several manifestations, encompassing hyperthyroidism, which denotes excessive thyroid activity, and hypothyroidism, which signifies insufficient thyroid function [2]. These illnesses may have significant ramifications for individuals' general health and well-being. The precise assessment of thyroid function is of utmost importance in identifying and treating thyroid disorders. Thyroid function tests (TFTs) encompass the evaluation of thyroid hormone levels, including thyroxine (T4) and triiodothyronine (T3), in addition to thyroid-stimulating hormone (TSH). These diagnostic tests offer vital insights into the physiological processes of the thyroid gland, enabling healthcare practitioners to make well-informed judgments regarding appropriate treatment strategies [3].

Precise thyroid function testing must be considered, as it serves as the basis for suitable therapeutic interventions. The occurrence of misdiagnosis or insufficient monitoring has the potential to result in unsatisfactory outcomes and jeopardize the well-being of patients [3]. Hence, healthcare practitioners must thoroughly comprehend the intricacies associated with thyroid function testing. This review aims to explore the complexities of thyroid disorders and highlight the crucial significance of precise thyroid function testing in their diagnosis and treatment [4]. Through an analysis of existing scholarly literature and research outcomes, this review aims to present a thorough and inclusive examination of the topic, elucidating developing patterns, obstacles, and progressions in thyroid diagnostics [4].

The scope of this examination encompasses a broader range of topics than the fundamental principles behind TFTs. The subject matter involves a comprehensive investigation into different thyroid problems, including their causes and the changing methods used for diagnosis [2-4]. Furthermore, the present review will examine the ramifications of thyroid dysfunction on several physiological systems and the general well-being of humans. Gaining a comprehensive understanding of thyroid diseases and the importance of accurate TFTs is essential for healthcare providers and patients who desire a more profound appreciation of their medical issues [5]. The primary objective of this review is to establish a connection between scientific knowledge and public awareness to promote a better-informed and empowered approach to thyroid health [5].

This review aims to make a scholarly contribution to the current literature on thyroid disorders and the precise assessment of thyroid function. To offer a comprehensive examination of the topic, this resource is a beneficial asset for healthcare professionals, researchers, and others interested in gaining a more profound comprehension of thyroid-related matters. This study aims to contribute to the existing discourse on thyroid health by synthesizing current literature and research findings, thereby increasing awareness on the subject.

## Review

Historical perspective

The historical context surrounding TFTs has witnessed notable advancements, which mirror the progressive comprehension of thyroid physiology and diagnostic methodologies [1]. The development of thyroid function testing has been influenced by scientific inquisitiveness, technical progress, and a persistent endeavor to achieve precision in evaluating thyroid well-being [1].

Evolution of Thyroid Function Testing

The inception of thyroid function testing can be attributed to the early 20th century, during which researchers commenced investigations into the metabolic activity of the thyroid gland [2]. In the early stages, conventional techniques such as palpation and clinical symptom observation were utilized to diagnose thyroid diseases. Nevertheless, more intricate methodologies have surfaced with the advancement of knowledge regarding thyroid function. The advent of radioimmunoassay (RIA) during the mid-20th century represented a significant milestone in the progression of thyroid function testing [3]. This methodology facilitated the meticulous quantification of thyroid hormones, thereby establishing a foundation for enhanced diagnostic precision. The precision and sensitivity of TFTs were further enhanced by subsequent breakthroughs in immunoassay technology, such as enzyme-linked immunosorbent assay (ELISA) [4].

Significant Developments in Thyroid Diagnostics

Numerous significant developments have had a crucial impact on the evolution of thyroid diagnostics. An example of a significant accomplishment in the field was the identification of thyrotropin-releasing hormone during the 1960s, which contributed to a more comprehensive comprehension of the regulatory processes governing the release of thyroid hormones [5]. This discovery established the basis for developing more precise and efficient diagnostic methodologies. The introduction of thyroglobulin tests during the 1970s provided a significant means of monitoring individuals with thyroid cancer following their therapy. The use of thyroglobulin testing in diagnostic methods has yielded substantial advancements in detecting recurring thyroid cancer, augmenting patient care, and overall results [6]. The arrival of molecular testing in thyroid diagnosis emerged during the 21st century. Identifying distinct genetic variants linked to thyroid malignancies has facilitated the advancement of molecular diagnostic assays, enhancing the precision of risk assessment and individualized therapeutic strategies for individuals exhibiting thyroid nodules [6].

Previous Challenges and Limitations

Although significant advancements have been made in thyroid function testing, it is essential to acknowledge the presence of certain obstacles and limitations. A notable constraint was the dependence on a solitary biomarker, such as TSH, to identify thyroid diseases [7]. Although this technique has demonstrated efficacy in numerous instances, it needs to be improved in comprehensively capturing the intricate nature of thyroid function, occasionally resulting in erroneous diagnoses. Another historical obstacle that was encountered pertained to the need for uniformity in TFTs, resulting in discrepancies in outcomes across diverse laboratory settings [7]. The International Federation of Clinical Chemistry and Laboratory Medicine (IFCC) has undertaken standardization initiatives to tackle this matter, aiming to enhance uniformity and dependability in thyroid function testing on a global scale. The early assays faced hurdles due to technological limitations, as several procedures were time-consuming and necessitated substantial sample volumes [8]. The introduction of automated platforms and high-throughput technologies has subsequently addressed numerous constraints, resulting in optimizing the testing procedure and enhancing productivity.

The historical context of thyroid function testing demonstrates a progression from basic clinical observations to advanced and precise diagnostic techniques. The field of thyroid diagnostics has witnessed significant advancements throughout its growth, characterized by notable breakthroughs that have contributed to a deeper comprehension of thyroid functionality and enhanced the precision of diagnostic methodologies [9]. Despite encountering various hurdles and limits, thyroid function testing has significantly progressed due to continuous research and standardization efforts. The historical background outlined here serves as a basis for understanding the present status of thyroid diagnostics and predicting future developments in pursuing improved and individualized methods for managing thyroid health.

Modern TFTs

The assessment of thyroid function has evolved significantly, progressing from basic clinical observations to a comprehensive range of advanced testing methodologies. A wide range of laboratory techniques marks the current landscape of thyroid health research, enhancing our extensive knowledge in this field [9]. As we explore the contemporary period of TFTs, it becomes apparent that progress in immunoassays, molecular testing, automation, and the investigation of innovative biomarkers are influencing the current and future landscape of thyroid diagnostics.

Examination of Contemporary Testing Approaches

Thyroid function testing has emerged as a fundamental component in diagnosing and treating thyroid problems within the current medical context. The critical variables evaluated in these examinations comprise TSH, T4, T3, and, in some instances, thyroglobulin [10]. TSH, the most sensitive marker for evaluating thyroid function, is frequently utilized to assess the overall thyroid status. The integration of TSH measurements with T4 and T3 levels offers a full assessment of thyroid activity [4].

Recent Developments in Laboratory Techniques

The utilization of immunoassays has been widely employed in the field of thyroid function testing due to its ability to provide high levels of sensitivity and specificity. The technique known as RIA, which emerged during the mid-20th century, was a groundbreaking method that enabled the highly accurate quantification of thyroid hormones [5]. Nevertheless, the apprehensions surrounding radiation exposure led to a transition towards non-radioactive substitutes. The ELISA has emerged as a viable alternative to RIA due to its comparable precision and enhanced safety profile. The utilization of enzyme-labeled antibodies in ELISA has been extensively embraced in thyroid diagnosis [6]. Recently, there has been an increased recognition of the significance of chemiluminescent immunoassays, which provide improved sensitivity and a broader spectrum of detection. The advancements in immunoassay technologies have substantially enhanced the precision and effectiveness of TFTs. Incorporating molecular testing into thyroid diagnosis signifies a significant change in the area. Molecular assays investigating genetic alterations linked to thyroid malignancies offer substantial contributions to risk assessment and the development of tailored treatment strategies [6-8]. Including distinct genetic alterations, such as BRAF and RAS mutations, has become essential in evaluating the malignancy risk associated with thyroid nodules. Molecular testing has a dual purpose in thyroid cancer, as it not only facilitates the identification of the disease but also provides valuable insights for guiding treatment choices and predicting patient outcomes [9].

Implementing automation has brought about a significant transformation in laboratory procedures, namely in thyroid function testing. Automated platforms have several benefits, including enhanced operational efficiency, decreased time required for completion, and mitigated potential for human mistakes [10]. These devices provide the concurrent analysis of several samples, increasing the efficiency of thyroid function testing in circumstances with high sample volumes. The incorporation of automation has optimized laboratory processes, guaranteeing consistent and replicable outcomes.

Novel Biomarkers and Precision Markers

The quest for enhanced accuracy in thyroid diagnosis has prompted researchers to investigate other biomarkers that extend beyond conventional thyroid hormones. Biomarkers such as thyroglobulin, calcitonin, and thyroid peroxidase antibodies have garnered considerable interest due to their potential to enhance diagnostic precision and offer supplementary clinical insights [11]. Precision markers, such as microRNA and circulating tumor DNA, are emerging as promising strategies for managing thyroid cancer. These indicators provide insight into the molecular composition of thyroid cancers, facilitating a more comprehensive comprehension of the biology of the illness. Integrating these factors into diagnostic algorithms presents the potential to improve diagnostic specificity and inform therapy choices [12].

The contemporary period of thyroid function assessments is distinguished by a wide range of advanced approaches that have significantly transformed the field of thyroid diagnostics [10]. The advancements in immunoassays, molecular testing, and automation have considerably enhanced the precision, efficiency, and depth of information produced by thyroid function testing. Investigating new biomarkers and precision markers signifies the continuous pursuit of a more refined and individualized approach to thyroid health [11]. With the ongoing evolution of technology, there is a promising outlook for the future in terms of more sophisticated and precise techniques, which will further enhance our capacity to comprehend and effectively handle thyroid problems comprehensively.

Precision diagnostics

Within the field of thyroidology, precision diagnostics has developed as a highly influential methodology, employing new technology to customize medicinal interventions with unparalleled accuracy [12]. The concept of a paradigm shift, frequently referred to as precision medicine, acknowledges individual patients' distinct molecular and genetic attributes, hence facilitating the development of tailored treatment approaches. As the multidimensional function of precision diagnostics in thyroidology is examined, it becomes apparent that this approach goes beyond traditional testing methods. It includes customized treatment techniques and the use of imaging and molecular diagnostics [13].

Significance of Precision Medicine in the Field of Thyroidology

Precision medicine, commonly referred to as personalized medicine, is centered on tailoring healthcare to individuals' unique characteristics, enabling more efficient and focused therapies. Precision diagnostics are of utmost importance in thyroidology as they are essential in comprehending the numerous complexities associated with thyroid illnesses [12]. This methodology recognizes the variability across individuals, including those with comparable clinical manifestations. The introduction of molecular testing has substantially impacted the field of thyroidology, enhancing the panorama of precision medicine. In contrast to exclusive dependence on conventional thyroid function testing, molecular diagnostics explore the genetic and molecular foundations of thyroid diseases [13]. In the context of thyroid cancer, molecular testing enables the identification of distinct genetic alterations such as BRAF and RAS. This process plays a crucial role in the classification of tumors. It provides valuable guidance for making informed decisions regarding treatment strategies [14].

Precision medicine in the field of thyroidology encompasses a comprehensive range of elements, including clinical, genetic, and environmental aspects, extending beyond the confines of the laboratory [9]. Doctors' utilization of genetic profiling permits the identification of inherent predispositions, hence facilitating a proactive and personalized approach to patient care. Precision medicine principles enable healthcare professionals to transcend a standardized system, recognizing the distinctive characteristics of individual patients' thyroid health [10].

Adapting Treatment Approaches According to Test Findings

Precision diagnostics' capacity to direct individualized treatment options is a distinguishing characteristic. The conventional method for managing thyroid diseases often entailed employing a standardized regimen grounded in broad diagnostic classifications. Precision medicine facilitates a more intricate comprehension of a patient's medical state, enabling personalized treatment approaches [15]. In thyroid cancer care, molecular testing plays a significant role in informing treatment options by enabling the detection of distinct genetic abnormalities. Individuals with specific genetic variants may benefit from tailored therapeutic interventions. However, other conventional methods, such as surgical procedures or radioactive iodine treatment, may be necessary for different patients [15]. Precision diagnostics play a crucial role in ensuring correct diagnosis and informing the choice of therapeutic measures, considering each case's distinct characteristics.

Precision diagnostics play a crucial role in the management of autoimmune thyroid disorders, including Hashimoto's thyroiditis and Graves' disease [16]. These advanced diagnostic techniques aid in identifying specific indicators, enabling healthcare professionals to customize treatment strategies accordingly. This may entail optimizing thyroid hormone replacement therapy or considering immunomodulatory medications based on the individual's immunological profile [16].

The convergence of imaging and molecular diagnostics has emerged as a significant area of medical research and development. The amalgamation of imaging and molecular diagnostics presents a collaborative strategy towards precision medicine in thyroidology [16]. Imaging modalities, such as ultrasound and nuclear medicine studies, offer significant anatomical and functional insights. Integrating molecular diagnostics with imaging modalities improves the accuracy of diagnosing and characterizing thyroid problems. Ultrasound imaging is a critical component in the evaluation of thyroid nodules [17]. The use of molecular testing in conjunction with ultrasound findings facilitates a more precise risk assessment, enabling differentiation between benign and malignant nodules. The utilization of this comprehensive methodology assists healthcare professionals in making well-informed judgments about the necessity of additional diagnostic procedures or surgical intervention [17].

In thyroid cancer, using positron emission tomography (PET) scans and other nuclear medicine imaging techniques might be advantageous in the localization of metastases and the facilitation of treatment planning. The imaging findings are complemented by molecular diagnostics, which offer valuable insights into the tumor's genetic characteristics [18]. This information plays a crucial role in determining the patient's prognosis and guiding the selection of appropriate targeted therapies. The integration of imaging and molecular diagnostics is a prime illustration of the comprehensive approach inherent in precision medicine within the field of thyroidology [19]. This approach facilitates a thorough evaluation of thyroid problems, considering both the structural and molecular components, to develop a customized and accurate treatment strategy.

Precision diagnostics has brought about a paradigm shift in thyroidology, revolutionizing the methods employed for diagnosing and treating thyroid-related conditions. The role of precision medicine extends from the laboratory to the clinic, encompassing genetic, molecular, and clinical factors [20]. By tailoring treatment approaches based on test results and integrating imaging with molecular diagnostics, healthcare practitioners can provide more accurate diagnoses and personalized interventions for patients with thyroid disorders. This evolving landscape promises continued advancements, further refining our ability to understand, diagnose, and manage thyroid conditions precisely [15].

Clinical implications of TFTs

Influence on Patient Management

TFTs significantly impact the management of patients with various thyroid diseases. TSH is frequently regarded as the fundamental component of these assays, as it plays a crucial role in regulating thyroid function. Deviant levels of TSH, whether raised or reduced, function as significant markers that trigger additional inquiry and subsequent intervention [21]. Hypothyroidism, which is defined as a thyroid gland that is not producing enough hormones, necessitates the commencement of thyroid hormone replacement therapy due to excessive levels of TSH. Levothyroxine, a synthetic derivative of T4, is frequently used to reestablish euthyroidism and mitigate the accompanying symptoms [21]. On the other hand, hyperthyroidism, which is characterized by excessive thyroid activity, necessitates interventions such as antithyroid medicines, radioactive iodine therapy, or surgical methods to restore normal thyroid function due to the lowered levels of TSH [22].

In addition to TSH, the evaluation of free T4 and T3 levels provides a thorough overview of thyroid functionality [22]. Healthcare practitioners utilize deviations from normal ranges to inform their decision-making process to refine treatment plans and make necessary adjustments to drug dosages, aiming to achieve optimal thyroid balance.

Risk Stratification and Prognostic Value

TFTs are essential in evaluating risk and prognosis, specifically thyroid nodules and thyroid cancer. The assessment of serum thyroglobulin, frequently combined with imaging examinations, assists in monitoring individuals with thyroid cancer following their therapy. Elevated or increasing levels of thyroglobulin can serve as an indicator of the presence of remaining or recurring disease, which can have an impact on decisions about additional treatment approaches [22]. TFTs, in conjunction with imaging modalities such as ultrasonography, contribute to the risk classification process in evaluating thyroid nodules. The categorization of nodules into low, intermediate, or high suspicion for malignancy is determined by the American Thyroid Association (ATA) recommendations, which incorporate a mix of clinical and laboratory characteristics. The risk categorization in question serves as a framework for determining the appropriate level of diagnostic interventions, which might vary depending on the severity of the condition [23]. These interventions may range from closely monitoring low-risk nodules to conducting fine-needle aspiration for high-risk lesions.

Difficulties Encountered During Clinical Implementation

Despite the unquestionable therapeutic usefulness of thyroid function testing, their application has ongoing hurdles. An area of considerable difficulty lies in interpreting findings within the framework of individual patient attributes. Various factors, including age, pregnancy, and specific drugs, can affect thyroid function, hence requiring a careful and nuanced approach when interpreting the results [21-23]. Furthermore, the continual problem of standardizing reference ranges among laboratories highlights the necessity of harmonization efforts to maintain uniformity in results. The complexities associated with autoimmune thyroid illnesses, including Hashimoto's thyroiditis and Graves' disease, introduce an additional level of intricacy [23]. The presence of varying antibodies and possible discrepancies between clinical observations and laboratory results necessitates a comprehensive approach to evaluating patients.

In addition, the clinical analysis of TFTs encompasses more than just numerical data. The comprehensive decision-making process necessitates the integration of patient symptoms, medical history, and imaging findings. Applying a comprehensive methodology is especially pertinent when thin-film transistors may not offer a definitive diagnostic representation [23]. The present study focuses on the exploration of emerging technologies in the field of thyroid diagnostics.

Next-Generation Sequencing in Thyroid Diagnostics

The introduction of next-generation sequencing (NGS) has brought about a significant transformation in the field of genetic testing. It shows considerable potential in the domain of thyroid diagnostics. NGS enables the concurrent examination of numerous genes, offering a comprehensive perspective on the genetic profile linked to thyroid illnesses, namely thyroid malignancies [24]. NGS enables the detection and characterization of distinct genetic alterations, such as BRAF and RAS, commonly present in thyroid malignancies. Using NGS technology in standard diagnostic practices improves the accuracy of risk assessment [24]. It provides valuable insights for making informed treatment choices. The utilization of NGS in deciphering the genetic composition of thyroid cancers enhances the individualized nature of medical interventions [25]. This technology aids physicians in making informed decisions on selecting targeted therapeutic strategies and accurately forecasting the potential outcomes of treatments.

Advancements in Point-of-Care Testing

The progress made in point-of-care testing (POCT) has increased the accessibility of thyroid diagnostics, allowing for expedited and convenient patient evaluations. POCT devices, which encompass a variety of tools such as handheld analyzers and lab-on-a-chip technologies, provide the immediate assessment of thyroid hormones and other pertinent biomarkers [26]. In situations when timely outcomes are of utmost importance, such as in emergency departments or outpatient facilities, POCT helps accelerate the process of diagnostic assessments. The importance of timely identification and intervention becomes especially significant in thyroid storm, a critical consequence of hyperthyroidism [26]. POCT developments have contributed to improved accessibility and efficiency. However, it is crucial to exercise caution and carefully evaluate the reliability and correctness of the results obtained by POCT, particularly when comparing them to standard laboratory-based assays [27].

Artificial intelligence (AI) applications are wide-ranging and have significant implications across various domains. AI has emerged as a transformative technology, revolutionizing industries and enabling new possibilities. The utilization of machine learning methods in AI applications is significantly transforming the field of thyroid diagnosis [27]. AI algorithms can evaluate extensive datasets, encompassing clinical, imaging, and molecular information, to extract patterns and insights that may be elusive to conventional analytical methods.

Within the domain of thyroid imaging, AI has exhibited the capacity to augment the precision of ultrasound interpretations, hence assisting in the characterization of thyroid nodules. Incorporating AI into the field of molecular diagnostics enhances the process of risk stratification by effectively detecting intricate patterns within genetic information [28]. Furthermore, using AI applications significantly promotes the standardization of result interpretation and decision-making processes. This, in turn, helps to address some of the difficulties that arise from the subjective nature of specific diagnostic assessments [27].

TFTs have significant clinical consequences since they are crucial in guiding patient management, aiding risk classification, and contributing to prognostic assessments. Despite the various difficulties encountered in the practical application of these methods, continuous endeavors toward standardization and adopting a comprehensive approach to interpretation improve their effectiveness [28]. The incorporation of nascent technologies, including NGS, breakthroughs in POCT, and applications of AI, signifies the advent of a novel epoch in precise thyroid diagnostics. These technologies enhance the precision of diagnostic procedures and facilitate the development of individualized and streamlined methods for managing thyroid problems [29]. The ongoing development of the discipline is expected to result in the future when there is a complete understanding and accurate management of thyroid health facilitated by the collaborative integration of established diagnostic techniques and developing technology [29].

Challenges and considerations in thyroid testing

Challenges and Pitfalls in the Interpretation Process

The assessment of thyroid function, although crucial for making informed clinical decisions, presents notable difficulties in interpreting test results. One significant obstacle is the diversity shown in individual reactions to thyroid disease. Various factors, including age, sex, and underlying medical problems, might influence thyroid hormone levels, introducing complexities in determining universally applicable reference ranges [30]. In addition, it is essential to consider the dynamic nature of thyroid physiology and the potential influence of non-thyroidal disorders when interpreting TFTs, as these factors might cause changes in hormone levels. When encountering subclinical thyroid dysfunction characterized by TFT, results deviate from the normal range [31]. However, with no apparent or mild symptoms, deciding to commence treatment becomes a complex and intricate deliberation. A meticulous evaluation of individual patient characteristics is necessary to carefully weigh the possible advantages of intervention against the hazards associated with overtreatment [5].

Furthermore, including antibodies in autoimmune thyroid illnesses introduces an additional level of intricacy. The correlation between TST outcomes and clinical manifestations may not consistently coincide, requiring a thorough assessment that integrates laboratory data and patient-reported symptoms [6]. The concept of standardization and harmonization refers to the process of establishing uniformity and consistency in various aspects within a given context. The continuous difficulty of achieving consistent thyroid testing among laboratories persists. Discrepancies in the findings of TFTs might arise due to variability in assay methodologies, reference ranges, and calibration procedures, compromising the consistency and comparability of such tests [9]. Standardization and harmonization measures are paramount in guaranteeing the reliability and significance of test outcomes, irrespective of the laboratory responsible for conducting the study.

Significant progress has been achieved by the IFCC and the ATA in the establishment of guidelines for the standardization of TFTs [10]. Nevertheless, the global implementation of these recommendations continues to be a subject of ongoing development. Addressing standardization difficulties is crucial to provide precise cross-comparison of outcomes, particularly in a global context where patients may access healthcare services across diverse healthcare systems [11].

Ethical and Legal Considerations Surrounding Precision Thyroid Diagnostics

Precision medicine's emergence in thyroid diagnostics necessitates a thorough investigation of the ethical and legal implications involved. Integrating genetic and molecular testing into patient care raises concerns about privacy, permission, and possible inadvertent findings [12]. The genetic testing of patients with thyroid diseases has the potential to provide additional information regarding their susceptibility to other medical conditions. This raises ethical concerns around the extent of consent required for such testing and the responsibility to disclose incidental discoveries [14].

In addition, the ethical quandaries arising from the possibility of genetic discrimination are worth considering. Patients may experience apprehension regarding the potential influence of gene information suggesting susceptibility to thyroid problems on their eligibility for insurance coverage or employment opportunities [15]. Establishing an ethical framework that is both intelligent and adaptable is necessary to strike a balance between the advantages of genetic insights and the imperative to safeguard patient privacy and prevent discriminatory practices. Legal considerations are a significant factor, especially in areas where rules about genetic testing and data protection are undergoing development [16]. The ethical landscape of precision thyroid diagnostics encompasses the crucial elements of patient knowledge, patient autonomy in genetic information control, and protection against potential damage or discrimination.

Future directions in thyroid testing: a prospective analysis

Promising Research Avenues

The field of thyroid testing is poised for significant advancements in the following years as ongoing research endeavors seek to improve diagnostic accuracy and deepen our comprehension of thyroid-related conditions. Current genomics research mainly elucidates the underlying genetic mechanisms associated with thyroid malignancies and autoimmune thyroid disorders [15]. Identifying supplementary genetic markers and comprehending their functional implications can facilitate the development of more precise and individualized strategies for diagnosis and therapy. Investigating innovative indicators beyond conventional thyroid hormones represents a potentially fruitful direction [17]. Proteomic and metabolomic studies can reveal distinct signatures linked to thyroid dysfunction, offering supplementary information layers to facilitate a more thorough evaluation [28].

Possible Advances

The field of thyroid diagnostics has the potential to experience significant advancements due to the continuous development of imaging technology. The objective of utilizing high-resolution imaging modalities, such as sophisticated ultrasound techniques and PET scans, is to enhance the characterization of thyroid nodules [25]. Integrating these technologies with molecular diagnostics promises to improve risk stratification and facilitate more precise therapeutic decision-making. In the field of laboratory testing, there are emerging assay techniques that exhibit enhanced sensitivity and specificity [26]. Technological advancements have the potential to facilitate the development of POCT tools, which can offer quick and accurate assessments of thyroid function. This is particularly beneficial in settings where resources are limited [29].

Implications of Personalized Medicine

The future of thyroid testing is closely interconnected with the broader framework of customized treatment. As the scope of our comprehension of individual genetic profiles and molecular signatures broadens, the practice of customizing interventions according to precise diagnostic information will increasingly become a norm [26]. The utilization of molecular testing, such as NGS, is anticipated to significantly impact the comprehensive understanding of the complex genetic characteristics associated with thyroid problems. The potential for individualized therapy is vast with AI in thyroid diagnostics [27]. AI systems, when trained on extensive datasets that include genetic, clinical, and imaging information, can detect subtle patterns that conventional analytical methods may not quickly identify [28]. This approach improves the precision of diagnostic assessments. It facilitates a more comprehensive comprehension of unique patient characteristics, informing tailored treatment approaches.

The complexities involved in interpreting thyroid testing findings, establishing standardized practices, and addressing ethical and regulatory aspects in the context of precision diagnostics highlight the challenges and issues associated with this field [29]. Notwithstanding these limitations, the future of thyroid testing is characterized by auspicious research pathways, prospective advancements in imaging and laboratory technology, and significant ramifications for customized therapy [30]. The continuous integration of scientific progress and ethical deliberations will play a pivotal role in shaping a future whereby thyroid diagnostics exhibit enhanced precision and are tailored to the individual attributes of each patient. As we embark on the path toward the end, our collective vision encompasses advancing patient outcomes, heightened accuracy in diagnostic procedures, and adopting a more individualized approach to managing thyroid health [31].

## Conclusions

In conclusion, our investigation into thyroid diagnostics has shed light on the complex issues surrounding the interpretation of results, the establishment of standardized procedures, and the ethical considerations involved. The intricate characteristics of thyroid problems, individual variances, and the complex nature of autoimmune conditions require a comprehensive approach to the diagnosis process. The continuous endeavors to establish uniformity in thyroid testing on a global scale and address the ethical considerations associated with precision diagnostics highlight the importance of maintaining a careful equilibrium between scientific advancement and the welfare of patients. In anticipation of future developments, the ongoing progress in genomic research, emerging biomarkers, and imaging technologies present promising opportunities to improve diagnostic accuracy and facilitate a future where thyroid health is comprehensively understood and personalized approaches are central to patient care.
